# Mechanisms of Host Behavioral Change in *Toxoplasma gondii* Rodent Association

**DOI:** 10.1371/journal.ppat.1004935

**Published:** 2015-07-23

**Authors:** Ajai Vyas

**Affiliations:** School of Biological Sciences, Nanyang Technological University, Singapore; University of Wisconsin Medical School, UNITED STATES

## Introduction

The behavioral manipulation hypothesis predicts that parasites can change host behavior in a way that benefits the parasites and not the host (extensively reviewed in [1–9]). In other words, the hypothesis predicts that genes of a parasite can produce an "extended" phenotype that manifests beyond a parasite's soma [10]. Protozoan parasite *Toxoplasma gondii* (henceforth toxoplasma) is an often-cited example. Chronic toxoplasma infection reduces aversion of rodents to cat odors, plausibly increasing predation by its definitive felid host [11]. Here, I enumerate main narratives that have emerged in the past decade about biological mechanisms of behavioral change in rodents after toxoplasma infection.

Cats are infected by toxoplasma when they eat infected prey. The parasite undergoes gametogenesis in cat intestines, resulting in eventual shedding of fecal oocysts that are ingested by intermediate hosts. Entry in the cat is important for the parasite because it permits a) sexual recombination; b) infection of herbivore hosts who otherwise cannot be infected through carnivory between intermediate hosts; and c) the discharge of highly infectious and resilient oocysts into the environment. Yet, entry of the parasite in the cat is constrained by predation rates. Preys of cats avoid cats and cat odors [12]. Apropos, toxoplasma infection leads to reduced aversion of rodents to cat odors [11]. A subset of animals also develops an atypical and “fatal” attraction [11,13]. These behavioral observations suggest, but do not prove, that the parasite creates an extended phenotype in the host behavior. The caution in the preceding sentence is necessary because it is yet unknown if infected rodents are indeed predated more frequently by cats.

Toxoplasma is also sexually transmitted through the male ejaculate in rats [14]. Apropos, male rats infected with toxoplasma become more attractive to females [15]. Uninfected females spend greater time near infected males and allow them greater reproductive access [14]. These observations suggest a second parasitic manipulation of the host behavior, whereby being infected creates greater avenues for sexual transmission of the parasite itself [9].

Biological pathways underlying mate choice and innate aversion to predator odor are relatively well-studied in rodents. This has allowed researchers to study proximate mechanisms of parasitic behavioral manipulation in greater detail in this association compared to other host–parasite relationships. This mechanistic research has focused on three main narratives.

## Narrative #1: Tropism to Specific Regions of the Brain

This narrative posits that toxoplasma preferentially concentrates in certain brain regions; and this tropism can explain host behavioral changes through local manipulation of neuronal signaling and/or damage. Toxoplasma exhibits a decided tropism to brain, testes, and eyes. These organs are immune-privileged, in the sense that immune cells have limited access to these sites. Some experimental evidence suggests that toxoplasma gains entry into these sites through brain endothelial cells [16] or by using dendritic immune cells as Trojan horses [17]. The parasite then forms bradyzoite-containing tissue cysts that undergo periodic cycles of rupture and encystment (i.e., recrudescence). Several studies have mapped sites of encystment within the brain, with the hope that location of these cysts has some bearing on the host behavior. Two earliest studies in this regard reported a rather widespread occurrence of tissue cysts in a variety of brain regions [13,18]. Both of these reports suggested a mild tropism to nucleus accumbens, ventromedial hypothalamus, or amygdala. These brain structures are involved in decision making and generation of fear [19,20]. Thus, the suggestion was that presence of cysts somehow compromised normal functioning of these brain regions and led to deficits in processing of fear or decision-making capacities. Yet, subsequent detailed analysis of cyst distribution failed to reveal substantial tropism in any of these three brain structures in mice and rats, instead reporting a rather “probabilistic” spread of the parasites [21]. Even more surprisingly, further experiments demonstrated that change in host behavior could be observed even after extensive clearance of parasite cysts within brain [22]. This suggests that toxoplasma tropism or lack of tropism does not have a causal relationship with the behavioral change. A nontropic model must thus be sought. Opinions still remain divided on this issue. For example, it has been suggested that within infected animals, those with presence of parasitic cysts within certain brain regions experience greater magnitude of behavioral change [23]. Another possibility in this regard is that toxoplasma could “coopt” brain cells without invading them [24]. The parasite is known to inject effector proteins inside host cells during early phases of invasion [24]. In many of the brain cells, the parasite injects these effector proteins but then does not, or fails to, gain residence. Such covert tropism will be difficult to detect by mere enumeration of parasite presence in various brain regions. Excitingly, new transgenic methods now allow visualization of “toxoplasma-kissed” neurons [25], although selective tropism of such events is presently unstudied.

## Narrative #2: Disruption of Dopamine Signaling in the Brain

An important candidate in the nontropic model is disruption of brain dopamine signaling. The genome of toxoplasma contains two genes (AAH1 and AAH2) that bear striking sequence similarity to a mammalian enzyme called tyrosine hydroxylase [26]. This enzyme in mammals catalyzes conversion of L-tyrosine to L-3,4-dihydroxyphenylalanine. This reaction is a rate-limiting step in synthesis of dopamine. While dopamine is often presented as the neurotransmitter that signals reward or pleasure, its more parsimonious role in the brain is in motivation and goal-directed behaviors [27]. The narrative then is that toxoplasma increases dopamine signaling in the host brain by virtue of supplying a rate-limiting enzyme for its synthesis [28,29]. This increase in dopamine signaling then interferes with host behavior, creating atypical motivation to explore predator odors. In support of this, drugs that interfere with the binding of dopamine to its receptors ameliorate effects of the infection on aversion to predator odors [30]. Tissue cysts within the infected brain contain high amounts of dopamine, and cultured dopaminergic cells secrete greater dopamine when infected with toxoplasma, suggesting the possibility of hyperdopaminergic drive in the infected brain [28]. This hypothesis does not necessarily require tropism of the parasite within brain regions that endogenously produce this neurotransmitter. A stochastic distribution of the parasite will provide tyrosine hydroxylase to a wide variety of brain regions. Yet, it is unlikely that nondopaminergic neurons will contain complementary enzymes required for dopamine synthesis. Moreover, any residual dopamine production in nonendogenous circuits is unlikely to have any effect because of lack of dopamine receptors in downstream efferent. In short, an intersection of generalized increase in tyrosine hydroxylase in brain with specific distribution of complementary proteins in endogenous dopaminergic circuit could create a rather specific behavior alteration. This hypothesis can reconcile lack of strong tropism of the parasite. Yet it obligatorily requires persistent presence of the parasite within brain. The relationship of dopamine alteration and increase in sexual attractiveness of infected males also remains unclear at present. A definitive proof of this hypothesis will require a future experiment with possible demonstration that disruption of parasitic tyrosine hydroxylase genes within brain necessarily results in loss of host behavioral change without affecting parasite survival itself.

Recently, a mutant parasite with ablation of one of the AAH genes (AAH2) has been described [29]. This ablation does not affect parasite viability, invasion, and transmission. It will be interesting to ask if infection with this mutant still causes host behavioral change. Contrary to predictions from dopaminergic mediation, infection with wild-type or ΔAAH2 mutant toxoplasma did not result in greater dopamine in mice brain. Similarly, overexpression of AAH2 genes in cell culture failed to augment dopamine content [29]. A double mutant parasite lacking both AAH genes has still not been successfully created.

## Narrative #3: Hormonal Upheavals and Concomitant Epigenetic Changes

An alternative nontropic model invokes parasite-induced tweaking of communication lines between brain and gonadal hormones. Toxoplasma invades rat testes upon infection, leading to a heavy cyst burden in epididymis and the ejaculates [14]. This is not surprising because the testes, like the brain, is an immune-privileged site. The infection results in up-regulation of testosterone synthesis within Leydig cells of the testes [31]. The narrative here posits that greater testosterone synthesis results in two simultaneous effects. One, it increases synthesis of male sexual pheromone by virtue of androgen dependence of these molecules [15]. Two, excess testosterone shifts the host towards sexual behaviors and away from defensive behaviors [32]. In support of this narrative, castrating male rats before infection prevents host behavioral change [31]. An important caveat in this experiment is the possibility that removal of testosterone by castration can potentially increase tonicity of the immune response during the acute phase of the infection, thereby changing the course of the infection. An unequivocal demonstration of the direct role of testosterone would require selective ablation of testosterone “increase” postinfection rather than removal of all testicular steroidogenesis preinfection.

This narrative nonetheless provides a plausible chain of events. In male rats, testosterone sustains synthesis of major urinary proteins in the liver. These proteins are necessary and sufficient to signal sexual attractiveness in a dose-dependent manner to females when eventually excreted in the urine [33]. Testosterone and/or its metabolic derivatives bind to their receptor found in the medial amygdala, a brain region involved in signaling presence of sexual opportunities [34,35]. This brain region contains population of neurons expressing arginine vasopressin, a neurotransmitter that mediates reproductive behaviors in many species (e.g., [36,37]). Male rats infected with toxoplasma exhibit reduced DNA methylation in promoter sites upstream of the arginine vasopressin gene in the medial amygdala, resulting in its greater production [32]. This is akin to observations noted during testosterone supplementation in uninfected rats [38]. These arginine vasopressin neurons are typically recruited during copulation or sensory stimulation by female presence in uninfected rats [37]. Atypically, the same population of neurons becomes activated during exposure to cat odors in infected male rats. Furthermore, pharmacological mimicry of this molecular change institutes decreased predator aversion akin to the effects of toxoplasma infection [32]. Thus, the proposal here is that the increase in testosterone synthesis mediates both aspects of behavioral manipulation of sexual attractiveness and reduction in aversion to cats. The presence of the parasite within the brain or its tropism to certain brain regions becomes merely incidental and non-necessary in this narrative.

The narrative detailed above encompasses substrates that are highly dimorphic between genders. Congruent to males, toxoplasma also reduces aversion to cat odors in female mice and rats [13,39]. Thus, proximate mechanisms involving gender-dimorphic biology needs to be yet reconciled with gender-nondimorphic behavioral effects of the parasitism. Mechanisms of the behavioral change in females are currently understudied, though preliminary evidence suggests changes in progesterone levels postinfection [39].

## Conclusions

Rats and mice infected with toxoplasma exhibit behavioral change in their aversion to cat odor and their sexual attractiveness to females. Previous work has resulted in three main classes of hypotheses pertaining to proximate mechanism of this phenomenon. Current work continues to test these hypotheses. More clarity about the mechanisms will plausibly inform ultimate causation of host behavioral change.
